# Evaluating probiotic efficacy on weight loss in adults with overweight through a double-blind, placebo-controlled randomized trial

**DOI:** 10.1038/s41598-023-45395-7

**Published:** 2023-10-24

**Authors:** Pernilla Danielsson, Resthie R. Putri, Claude Marcus, Emilia Hagman

**Affiliations:** Division of Pediatrics, Department of Clinical Science, Intervention and Technology, Karolinska Institutet, Solna, Sweden

**Keywords:** Microbiota, Randomized controlled trials

## Abstract

The aim was to assess the weight-reducing effects of various doses of a probiotic dietary supplement and evaluate the tolerance and safety of increased dosage. A 3-month double-blinded, randomized, placebo-controlled trial, followed by a 3-month open phase, was conducted at Karolinska Institutet, Sweden. The probiotic compound AB001 was tested at two doses (single and double) and compared with placebo during the blinded phase, and at triple dose during the open phase. Eighty-one volunteers, 18–45 years old, with overweight were included. The primary outcome was change in weight. Secondary outcomes were changes in; BMI, waist circumference, blood pressure, blood lipids, glucose metabolism, liver enzymes, vitamin levels, and bowel habits. After 3 months (n = 81), no difference in weight, BMI, waist circumference, blood pressure, or biomarkers were observed between the groups. Forty-five individuals continued with triple dose. The group with initial single dose decreased 0.93 ± 4.73 kg (p = 0.34), and the group with double dose initially decreased 1.93 ± 3.70 kg (p = 0.027). Reported changes in bowel habits and gastro-intestinal problems were similar for all doses. The results indicate that a long-term use of at least double dose AB001 may be more beneficial for weight loss than lower doses. However, in the double blinded phase, no differences between groups were found. The probiotic compound AB001 was well tolerated and can safely be used up to double dose for 90 days followed by triple dose for 90 days.

**Trial registration**: Clinicaltrial.gov NCT04897698, registered on 21 May 2021.

## Introduction

Based on the latest estimates and according to the World Health Organization, overweight and obesity affects almost 60% of adults in the European Union countries^1^. In the US the situation is even more severe, with an estimated prevalence of overweight and obesity of 74% among adults^2^. Overweight and obesity are responsible for causing at least 2.8 million premature deaths worldwide each year^3^.

Weight loss regimes rely on creating an energy deficit—where energy intake is lower than energy expenditure. However, this approach proves challenging for many individuals to effectively put into practice. Even though there are effective weight loss treatment such as pharmacological treatment and surgery for individuals with obesity, persons with overweight are usually deemed to handle their overweight themselves despite that overweight also is associated with long-term health hazards^4^. Hence, safe and well tolerable food supplements may be one way to further improve the effect of lifestyle modification both for treatment of obesity and for obesity prevention in already overweight subjects.

In humans, individuals with overweight exhibit a decreased gut bacterial diversity and an altered composition of bacterial species^5,6^. *Bacteroidetes* and *Firmicutes* are the two predominant phyla in human microbiota and an altered ratio of these is associated with several diseases, e.g. obesity is associated with a decrease in *Bacteroidetes* and an increase in *Firmicutes*^7^. Since the link between metabolic health and gut microbiota was discovered, there has been increasing interest in exploring the potential use of biological agents such as probiotics as an active therapeutic or preventive strategy in managing metabolic diseases. A meta-analysis revealed that certain species, namely *Lactobacillus gasseri* and *Lactobacillus plantarum*, exhibit positive effects on weight loss, while other species in the *Lactobacillus* genus, such as *Lactobacillus acidophilus*, *Lactobacillus fermentum*, and *Lactobacillus ingluviei*, are associated with weight gain^8^. Additionally, evidence indicate that probiotics may modify the secretion of hormones, neurotransmitters, and inflammatory factors, thereby preventing triggers for excessive food intake that could contribute to weight gain^9,10^. Further, *Bacillus subtilis* and *Bacillus coagulans* are species that have exhibited favorable effects on obesity-related inflammation, metabolic disturbances, and weight control in rodent models featuring obesity^11–13^. The full mechanism of probiotics on weight is unclear, but may involve altered metabolism of short-chain fatty acids in the gut and reduced leakage of proinflammatory molecules^14^. Nevertheless, the spectra of available probiotics in the open market is extensive, and therefore, this study aimed to investigate whether different doses of the probiotic supplement AB001 can reduce weight and if an increased dose of AB001 is tolerated and safe. Secondary aims were to study changes in; BMI, waist circumference, blood pressure, blood lipids, glucose metabolism, liver enzymes, vitamin levels, and bowel habits.

We hypothesized that probiotics would yield a greater weight loss than placebo and that there would be a dose-dependent relationship between the dose of probiotics consumed and weight loss.

## Methods

### Design

This was a single-center, double-blind, three parallel group, block randomized, placebo-controlled trial, followed by an open phase conducted at Karolinska Institutet, Karolinska University Hospital Huddinge, Stockholm, Sweden, between October 2021 and February 2022. The study was conducted according to the Declaration of Helsinki and ethically approved by the Swedish Ethical Review Authority in May 2021 (file no. 2021-02383 with amendment no. 2021-06174-02) and registered at ClinicalTrials.gov (NCT04897698) on 21/05/2021. The study followed CONSORT guidelines for trials.

### Subjects

Voluntary individuals who were 18–45 years old and met the criteria of BMI 26.0 to less than 30 kg/m^2^ and were willing to lose weight were eligible. Exclusion criteria was active weight loss in the last 3 months, the desire for or planned pregnancy upcoming months, chronic somatic diseases that may affect metabolic and/or intestinal function (e.g. diabetes, hypertension, dyslipidemia, Irritable Bowel Disease, gluten intolerance, pancreatic dysfunction, other conditions of malabsorption, neoplastic disease), allergies with previous anaphylactic reactions, abdominal surgery within 6 months prior to inclusion, current or history of eating disorders, extreme or unusual diets for the last 3 months which the investigator considered negatively could affect the outcome of the study (e.g. ketogenic diet and intermittent fasting), psychiatric disorders (e.g., schizophrenia, and other diagnoses that may influence compliance), drug or alcohol abuse, pharmacological treatment that may influence the study outcomes (e.g. systemic corticosteroid treatment and anti-obesity treatment, but not well-controlled treatment for hyperthyroidism), present or recent usage of other probiotic agents, and other conditions which the investigator considers could negatively affect the outcome of the study or study compliance (e.g. social vulnerability such as homelessness).

Participants were provided economic compensation for their participation.

### Tested compound

Dietary supplement consisting of capsules withholding bacteria with probiotic properties called AB001 (de Faire Medical AB, Stockholm Sweden). It contains strains of *Bacillus subtilis* (LMG P-32899) and *Bacillus coagulans* (LMG P-32921) with a concentration of 13 million colony forming units (cfu) per gram. Other ingredients (including stabilization) include fermented rice bran which has prebiotic properties, L-Cysteine, and Dextrin. The capsule shell is made of vegetable hydroxypropyl metylcellulosa (HPMC). Each capsule has a weight of 0.4 g, corresponding to 5.2 million cfu.

Due to ethical reasons, all participants received a pamphlet with general advice regarding recommended food and eating pattern based on Swedish guidelines.

### Procedures

If inclusion criteria were fulfilled, no exclusion criteria were identified, and written informed consent was obtained, the individual was included in the study. Participants were randomized to (a) double dose probiotics (four capsules probiotic per day), (b) single dose probiotics (two capsules probiotic per day and two capsules of placebo), or (c) placebo (four capsules of placebo). At the 3-month visit, participants who had single or double dose probiotics were offered to continue the study for three additional months, an open phase, with triple dose probiotics (six capsules per day).

**Figure 1:**
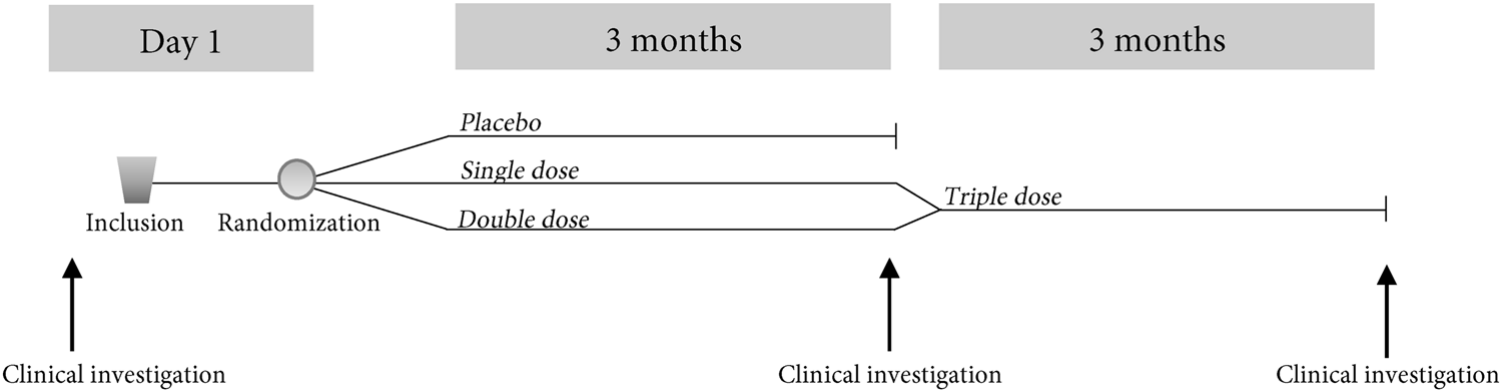
Schematic drawing of the study design.

Study participants were instructed to take half of the dose before breakfast and half of the dose before dinner each day. A schematic figure of the study process is shown in Fig. 1.

Clinical investigation was scheduled at baseline (date of inclusion), at 3-month follow-up (90 days after inclusion), and at 6-month follow-up (180 days after inclusion). At baseline, all participants filled in a questionnaire including demographics, well-being, and gastrointestinal function and habits, see Supplementary file S1. During the intervention, study participants were given access to a digital research support system (Evira AB, Stockholm, Sweden) consisting of a mobile phone application and a digital scale connected with Bluetooth to the app^15^. The app was connected to a database wherefrom the study coordinator could follow the study participants and communicate with them. Participants were given weekly questionnaires in the app regarding adherence to intervention, potential adverse events, well-being, changes in the daily life of diet, physical activity, and alcohol consumption, see Supplementary file S2. Further, they were instructed to weigh themselves at least weekly on the scale connected to the application in the phone. Further, all study participants had scheduled personal contact with the study coordinator via the app at week 2, 4, 8, 14, 16, and 20.

### Randomization and masking

Participants were randomly assigned (1:1:1) to single dose probiotics, double dose probiotics, or placebo using a fixed block size of nine participants and stratified for sex. Participants were randomized to (a) four capsules of probiotics per day or (b) two capsules of probiotics per day and two capsules of placebo, or (c) four capsules of placebo. Hence, all participants were to take four capsules per day, two before breakfast and two before dinner. The funder, which produced the product, packed the probiotics and placebo into pouches for morning and evening respectively, and labeled them group A–B (morning and evening), C–D, and E–F. At delivery of the study product, the labeling key was in a sealed envelope and kept in a locked space at the study center. The study was blinded during the first phase for both the researchers and the study participants. Type of received treatment was revealed to researcher and study participant at the 3-month follow-up, before statistical analyses. During the second phase of study, all participants received six capsules of probiotics per day (triple dose). This phase was non-blinded. Any participant that required extra capsules of probiotics/placebo due to prolonged follow-up time (e.g. due to current Covid-19 restrictions) were provided so by regular mail.

#### Clinical investigation

Clinical investigation was performed at baseline and at 3-month, and 6-month follow-up and included a medical examination that included cardiorespiratory and abdominal examinations was performed. In addition, weight was measured with a digital scale (Tanita BWB-800, Tanita Corp., Japan), height was measured with a wall-mounted stadiometer (SECA model 264, Seca, Germany) (only at baseline), waist circumference was measured using a measuring tape, and blood pressure was measured with a digital automatic monitor (Omron M3 Comfort, Omron, Japan).

#### Blood samples

Clinical laboratory tests were drawn at fasting state at baseline, 3-month follow-up, and 6-month follow-up and included; leukocyte concentration, MCH, platelets, mean cell [erythrocytes] volume (MCV), erythrocyte concentration, erythrocyte volume fraction (EVF), hemoglobin, high-sensitivity C-reactive protein (hs-CRP), glucose, hemoglobin (Hb) A1C, insulin, total cholesterol, triglycerides, low-density lipoprotein (LDL) cholesterol, and high-density lipoprotein (HDL) cholesterol, alanine aminotransferase (ALT), retinol (vitamin A), 25-hydroxyvitamin D, Tocopherol (vitamin E), thyroid stimulating hormone (TSH), thyroxine (T4), and triiodothyronine (T3). All blood samples were analyzed at Karolinska University Laboratory, Sweden.

Occasionally missing data of biomarkers occurred due to that blood samples were compromised at the laboratory, preanalytical factors such as hemolysis, or blood sample volume was not enough to be analyzed. Also, two high values of CRP, due to confirmed recent infection (Covid-19 and sinusitis) of 25 and 30 mg/L, were excluded from statistical analyses.

### Statistical analyses

#### Power calculation

Sample size calculation for this three-armed placebo-controlled study is performed in STATA software based on alpha = 0.0500 and power = 0.8000. Estimated change in body weight; Mean change in the placebo group = 0.00 kg, Mean change in single dose probiotic group = − 2.00 kg, Mean change in double dose probiotic group = − 4.00 kg. A within-group variance of 18.3 was anticipated. Depending on the estimated standard division within each group, 20–24 participants are needed per group, which means a total number of 60–72. We assumed a dropout rate of about 4–6 participants per group and therefore aimed to include 75–90 participants in total for randomization.

### Descriptive and outcome statistics

Data was checked for normality and presented with mean and standard deviation or median and interquartile range whenever appropriate. Descriptive data are presented with both measures with a marking of whether data was considered as normally distributed or not. Categorical variables are presented with frequencies and proportions.

Primary and secondary aims were investigated by comparing absolute changes between groups. Also, within group changes were analyzed, hence data was stratified by group. The results were based on observed cases and measures only. Hence, no data was imputed.

Unadjusted analyses were performed with t-test or ANOVA for normally distributed variables. Multiple comparisons were handled with Bonferroni correction. Wilcoxon test and Kruskal–Wallis were used as non-parametric test. To perform analysis of the inflammatory marker CRP, levels that were denoted at “< 1” from the laboratory was assigned a value of 0.5.

All analyses other than sample-size calculations were performed using SAS statistical software (version 9.4, Cary, North Carolina). All P-values presented were two-sided and a p-value less than 0.050 was denoted statistically significant.

### Role of funding

The funder, de Faire Medical AB (Sweden), manufacturer of the studied probiotic AB001 and provided matching placebo at no cost. Further, the funder provided Karolinska Institutet financial compensation for study-related expenses such as costs for analyses of blood samples and study subject reimbursement. The funder of the study had no role in study design, data collection, data analysis, data interpretation, or writing of the report. The corresponding author had full access to all the data in the study and had final responsibility for the decision to submit for publication.

## Results

Of the 81 included participants (57% females), the average age was 33.2 years, weight 83.9 kg, BMI 28.3 kg/m^2^. The majority (94%) were employed or students and 68% were born in Sweden. The demographic and baseline characteristics for each study group, and in total, are provided in Table 1.

**Table 1:** Demographic characteristics and baseline measures of included study individuals, n = 81.

		Total n = 81	Placebo n = 27	Single dose probiotics n = 27	Double dose probiotics n = 27	p between groups
Sex	% females	56.8	55.6	55.6	59.3	0.95^†^
Age (years)	Mean (SD)	33.2 (6.7)	32.2 (6.9)	35.8 (6.3)	31.4 (6.3)	0.034^†^
Weight (kg)	Median (IQR)	83.9 (13.0)	84.7 (9.4)	81.5 (20.2)	82.0 (19.7)	0.27^‡^
Height (cm)	Median (IQR)	171.0 (14.2)	172.1 (8.0)	168.9 (15.5)	169.0 (18.8)	0.33^‡^
BMI	Median (IQR)	28.3 (2.0)	29.9 (2.1)	28.2 (2.0)	28.2 (1.7)	0.10^‡^
Waist circumference (cm)	Mean (SD)	98.6 (7.0)	100.4 (5.8)	97.4 (6.6)	98.3 (8.3)	0.33^†^
Systolic blood pressure (mmHg)	Mean (SD)	129.0 (14.7)	129.0 (11.8)	131.8 (12.9)	126.4 (18.7)	0.41^†^
Diastolic blood pressure (mmHg)	Mean (SD)	81.9 (8.8)	81.0 (7.8)	83.7 (7.7)	81.0 (10.5)	0.45^†^
Nationality	% Swedish	67.9	74.1	51.9	77.8	0.088^¤^
Level of physical activity	Seldom/Sometimes/Often	12.3/55.6/32.1	3.7/40.7/55.6	22.2/59.3/18.5	11.1/66.7/22.2	0.014^¤^
Tobacco/nicotine usage	Never/Sometimes/Daily	75.3/16.1/8.6	88.9/7.4/3.7	66.7/18.5/14.8	70.4/22.2/7.4	0.29^¤^
Leukocytes (× 10^9^/L)	Mean (SD)	5.9 (1.5)	5.6 (1.0)	6.0 (1.6)	6.1 (1.8)	0.53^†^
Erythrocytes (× 10^12^/L)	Mean (SD)	4.8 (0.5)	4.8 (0.5)	4.9 (0.6)	4.7 (0.5)	0.41^†^
Hemoglobin (g/L)	Mean (SD)	141 (12)	142 (13)	141 (11)	140 (12)	0.78^†^
Hematocrit	Mean (SD)	0.42 (0.03)	0.43 (0.04)	0.42 (0.03)	0.42 (0.03)	0.58^†^
Mean corpuscular volume (fL)	Mean (SD)	88 (5)	89 (4)	87 (7)	89 (3)	0.20^†^
Mean corpuscular hemoglobin (pg)	Mean (SD)	30 (2)	30 (2)	29 (3)	30 (1)	0.31^†^
Thrombocytes (× 10^9^/L)	Mean (SD)	258 (56)	266 (60)	249 (44)	259 (62)	0.54^†^
Total cholesterol (mmol/L)	Mean (SD)	4.63 (0.78)	4.46 (0.84)	4.83 (0.81)	4.61 (0.67)	0.22^†^
LDL (mmol/L)	Mean (SD)	2.63 (0.67)	2.56 (0.71)	2.73 (0.68)	2.50 (0.64)	0.60^†^
HDL (mmol/L)	Mean (SD)	1.47 (0.43)	1.44 (0.32)	1.49 (0.53)	1.49 (0.42)	0.87^†^
Triglycerides (mmol/L)	Median (IQR)	0.93 (0.85)	0.88 (0.69	1.10 (1.26)	0.91 (0.95)	0.29^‡^
Fasting glucose (mmol/L)	Mean (SD)	5.33 (0.34)	5.28 (0.33)	5.37 (0.33)	5.34 (0.36)	0.63^†^
Fasting insulin (mIU/L)	Median (IQR)	9.10 (5.40)	7.85 (5.40)	9.20 (5.60)	9.60 (3.70)	0.53^‡^
HbA1c (mmol/mol)	Median (IQR)	34.0 (4.0)	34.0 (5.0)	33.0 (4.0)	33.0 (3.0)	0.67^‡^
ALT (µkat/L)	Median (IQR)	0.32 (0.21)	0.33 (0.29)	0.34 (0.14)	0.28 (0.17)	0.75^‡^
hsCRP (mg/mL)	Median (IQR)	1.00 (1.50)	1.00 (2.50)	1.00 (1.50)	0.50 (1.50)	0.38^‡^
Vitamin D (nmol/L)	Mean (SD)	59.6 (20.4)	62.1 (21.7)	54.3 (20.6)	62.5 (18.6)	0.25^†^
Vitamin E (µmol/L)	Mean (SD)	32.9 (6.7)	31.0 (5.1)	36.0 (8.4)	31.7 (5.0)	0.0096^†^
Vitamin A (µmol/L)	Mean (SD)	1.98 (0.45)	1.94 (0.44)	2.06 (0.43)	1.96 (0.48)	0.60^†^

^†^Parametric test: ANOVA.

^‡^Non-parametric test: Kruskal–Wallis.

^¤^χ^2^-test.

Generally, the randomization worked well, and no differences in sex, weight, BMI, waist circumference and blood pressure at baseline were observed. However, the group with single dose probiotics were on average 4.4 years older than the placebo group, p = 0.034. Further, the baseline levels of all biomarkers were similar across the groups except for vitamin E, where the placebo group had 4.3–5.0 µmol/L higher levels compared with single dose and double dose probiotics respectively. Over the study period, 1596 questionnaires were received. During the first 3 months 1033 questionnaires were received from 81 participants, and during months four through six, 563 questionnaires were received from 45 participants.

### Adherence

All 81 included participants remained to the 3-month follow-up. At the 3-month follow-up, 54 participants identified from the single or double dose of probiotic groups in the blinded phase were asked to continue with a higher dose of probiotics. Six participants declined further participation, two participants started the second phase, but dropped out, and one participant was excluded due to adverse event (obstipation concurrent with vomiting and gastrointestinal pain). Hence, 45 participants attended the 6-month follow-up visit.

Weekly electronic questionnaires asked if capsules had been missed, and if so, how many. During the first 3 months, all participants responded to at least 11 out of 12–14 weekly questionnaires (depending upon follow-up date). During months four through six, all participants responded to at least 11 out of 12–14 weekly questionnaires (depending upon follow-up date). Responses are presented at an aggravated level in Table 2.

**Table 2:** Reported adherence to intervention.

	Number (%) of responses not taking all capsules last week	Number of responses of how many capsules were missed during the last week		
		Less than 5 capsules	5–10 capsules	More than 10 capsules
Blinded phase				
Placebo group	37/345 (10.7%)	35	2	0
Single dose probiotic group	75/345 (21.7%)	63	8	4
Double dose probiotic group	61/343 (17.8%)	53	8	0
Open phase				
Initial Single dose probiotic group	81/298 (27.2%)	56	23	2
Initial Double dose probiotic group	41/265 (15.5%)	28	12	1

### Primary aim

The median (range) 3-month follow-up time was 91 (87–105) days, and the median 6-month follow-up was 182 (178–195) days. The longer follow-up time for some participants was due to current Covid-19 restrictions.

On average, all studied participants (n = 81) lost 0.6 kg in weight during the blinded 3-month phase. There was no difference in weight changes between the investigated groups (p = 0.89), Table 3. Analyses within groups revealed that none of the groups changed their weight statistically significant from baseline to follow-up, all p > 0.05.

**Table 3:** Unadjusted analyses of 3-month changes from baseline of primary and secondary outcomes of completed study individuals, n = 81.

		Total n = 81	Placebo n = 27	Single dose probiotics n = 27	Double dose probiotics n = 27	P between groups
Change in weight (kg)	Mean (SD)	− 0.63 (2.87)	− 0.84 (3.15)	− 0.52 (2.70)	− 0.54 (2.57)	0.89^†^
Change in BMI	Mean (SD)	− 0.22 (0.95)	− 0.92 (1.10)	− 0.17 (0.88)	− 0.20 (0.89)	0.89^†^
Change in waist circumference (cm)	Mean (SD)	− 1.1 (3.7)	− 1.8 (3.7)	− 1.1 (3.4)	− 0.41 (4.02)	0.41^†^
Change in systolic blood pressure (mmHg)	Mean (SD)	− 5.0 (8.9)	− 4.5 (6.6)	− 5.6 (8.5)	− 4.8 (11.2)	0.91^†^
Change in diastolic blood pressure (mmHg)	Mean (SD)	− 5.0 (6.7)	− 4.5 (6.2)	− 4.7 (6.0)	− 5.8 (7.8)	0.76^†^
Change in leukocytes (× 10^9^/L)	Mean (SD)	− 0.2 (1.4)	0.2 (1.1)	0.2 (1.1)	− 0.4 (1.4)	0.21^†^
Change in erythrocytes (× 10^12^/L)	Mean (SD)	− 0.0 (0.2)	− 0.1 (0.3)	− 0.0 (0.2)	− 0.0 (0.2)	0.67^†^
Change in hemoglobin (g/L)	Mean (SD)	− 0.4 (6.0)	− 1.6 (6.6)	0.3 (6.2)	0.2 (5.1)	0.43^†^
Change in hematocrit	Mean (SD)	− 0.0 (0.0)	− 0.0 (0.0)	− 0.0 (0.0)	− 0.0 (0.0)	0.76^†^
Change in mean corpuscular volume (fL)	Mean (SD)	− 0.1 (1.4)	− 0.1 (0.7)	− 0.0 (1.6)	− 0.2 (1.2)	0.92^†^
Change in mean corpuscular hemoglobin (pg)	Mean (SD)	− 0.1 (1.3)	− 0.1 (0.7)	− 0.3 (2.1)	0.1 (0.7)	0.65^†^
Change in thrombocytes (× 10^9^/L)	Mean (SD)	− 4 (35)	− 2 (38)	− 0 (22)	− 9 (43)	0.62^†^
Change in total cholesterol (mmol/L)	Mean (SD)	− 0.11 (0.50)	− 0.05 (0.40)	− 0.07 (0.48)	− 0.20 (0.60)	0.51^†^
Change in LDL (mmol/L)	Mean (SD)	0.02 (0.42)	0.02 (0.36)	0.06 (0.35)	− 0.03 (0.54)	0.67^†^
Change in HDL (mmol/L)	Mean (SD)	− 0.08 (0.19)	− 0.10 (0.14)	− 0.04 (0.18)	− 0.11 (0.23)	0.37^†^
Change in triglycerides (mmol/L)	Mean (SD)	− 0.13 (0.63)	0.03 (0.54)	− 0.21 (0.70)	− 0.20 (0.64)	0.29^†^
Change in fasting glucose (mmol/L)	Mean (SD)	− 0.03 (0.34)	0.01 (0.34)	− 0.10 (0.35)	− 0.02 (0.35)	0.46^†^
Change in HbA1c (mmol/mol)	Mean (SD)	− 0.16 (1.44)	0.04 (1.45)	− 0.30 (1.27)	− 0.22 (1.60)	0.67^†^
Change in fasting insulin (mIU/L)	Mean (SD)	− 0.38 (4.39)	− 0.52 (4.60)	− 0.11 (4.96)	− 0.53 (3.66)	0.93^†^
Change in ALT (µkat/L)	Mean (SD)	0.03 (0.23)	0.01 (0.19)	0.05 (0.20)	0.02 (0.28)	0.79^†^
Change in hsCRP (mg/L)	Median (IQR)	0.00 (1.00)	0.00 (1.50)	0.00 (0.00)	0.00 (1.00)	0.53^‡^
Change in vitamin A (µmol/L)	Mean (SD)	0.00 (0.37)	0.06 (0.25)	− 0.04 (0.45)	− 0.01 (0.39)	0.58^†^
Change in vitamin E (µmol/L)	Mean (SD)	0.95 (5.03)	1.74 (5.62)	0.89 (4.96)	0.22 (4.53)	0.55^†^
Change in vitamin D (nmol/L)	Mean (SD)	4.56 (19.55)	− 0.56 (13.04)	8.89 (20.27)	5.04 (23.40)	0.20^†^

^†^Parametric test: ANOVA.

^‡^Non-parametric test: Kruskal–Wallis.

**Figure 2:**
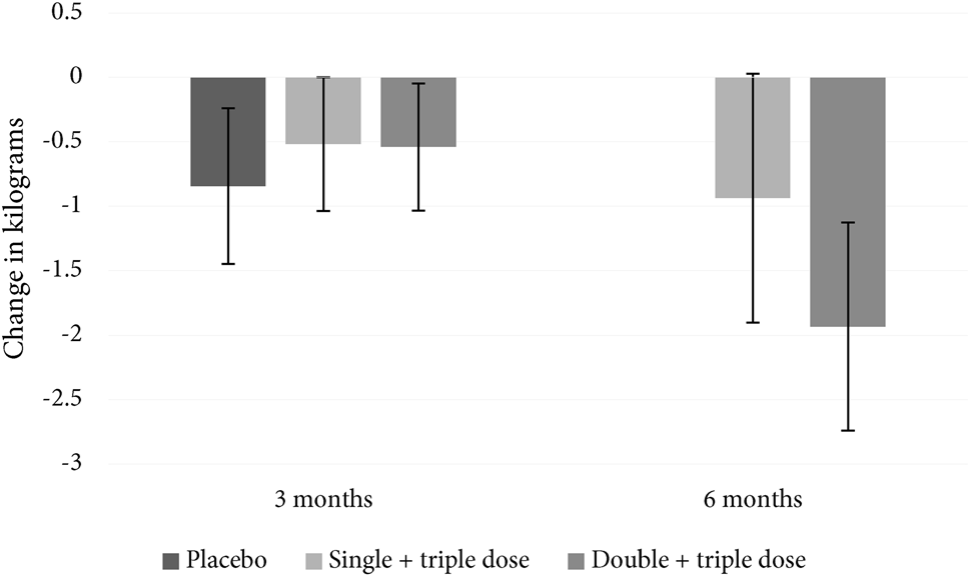
Average change in weight from baseline stratified by group. Error bars represent standard error.

After 6 months, the participating individuals (n = 45) had an average weight loss from baseline of 1.4 kg, p = 0.033, Table 4. When investigating each arm separately the 6-month weight loss in the group with single + triple dose probiotics (n = 24) was 0.94 kg, which was not a statistically significant weight loss from baseline, p = 0.34. However, the group that received double + triple dose probiotics (n = 21 47% of the available participants) had a statistically significant weight loss from baseline of 1.93 kg, p = 0.027, Fig. 2. There was no difference in weight changes between the investigated groups after 6 months (p = 0.44), Table 4.

**Table 4:** Unadjusted analyses of 6-month changes from baseline of primary and secondary outcomes of study individuals, n = 45.

		Total n = 45	Initial single dose probiotics n = 24	Initial double dose probiotics n = 21	P between groups	P total from baseline
Change in weight (kg)	Mean (SD)	− 1.40 (4.26)	− 0.93 (4.73)	− 1.93 (3.70)	0.44^†^	0.033^†^
Change in BMI	Mean (SD)	− 0.48 (1.40)	− 0.30 (1.49)	− 0.67 (1.29)	0.38^†^	0.027^†^
Change in waist circumference (cm)	Mean (SD)	− 2.8 (5.7)	− 2.90 (5.41)	− 2.78 (6.22)	0.95^†^	0.002^†^
Change in systolic blood pressure (mmHg)	Mean (SD)	− 6.9 (9.6)	− 8.4 (9.6)	− 5.2 (9.7)	0.28^†^	< 0.0001^†^
Change in diastolic blood pressure (mmHg)	Mean (SD)	− 5.8 (6.1)	− 6.3 (5.4)	− 5.3 (6.9)	0.60^†^	< 0.0001^†^
Change in leukocytes (× 10^9^/L)	Mean (SD)	− 0.3 (1.4)	− 0.3 (1.6)	− 0.4 (1.2)	0.71^†^	0.13^†^
Change in erythrocytes (× 10^12^/L)	Mean (SD)	− 0.1 (0.2)	− 0.1 (0.2)	− 0.1 (0.2)	0.18^†^	0.0018^†^
Change in hemoglobin (g/L)	Mean (SD)	− 2 (6)	− 4 (6)	0 (6)	0.030^†^	0.034^†^
Change in hematocrit	Mean (SD)	− 0.01 (0.02)	− 0.01 (0.02)	− 0.01 (0.02)	0.19^†^	0.0032^†^
Change in mean corpuscular volume (fL)	Mean (SD)	− 0.02 (1.47)	− 0.13 (1.69)	0.10 (0.21)	0.61^†^	0.92^†^
Change in mean corpuscular hemoglobin (pg)	Mean (SD)	0.2 (0.9)	0.1 (0.9)	0.4 (0.9)	0.26^†^	0.096^†^
Change in thrombocytes (× 10^9^/L)	Mean (SD)	− 3.6 (39.3)	− 2.1 (29.5)	− 5.3 (48.9)	0.81^†^	0.55^†^
Change in total cholesterol (mmol/L)	Mean (SD)	− 0.03 (0.64)	− 0.18 (0.66)	0.14 (0.60)	0.10^†^	0.75^†^
Change in LDL (mmol/L)	Mean (SD)	0.11 (0.60)	0.03 (0.60)	0.20 (0.59)	0.37^†^	0.23^†^
Change in HDL (mmol/L)	Mean (SD)	− 0.03 (0.26)	− 0.08 (0.31)	0.02 (0.17)	0.19^†^	0.46^†^
Change in triglycerides (mmol/L)	Mean (SD)	− 0.22 (0.67)	− 0.27 (0.73)	− 0.16 (0.61)	0.61^†^	0.036^†^
Change in fasting glucose (mmol/L)	Mean (SD)	− 0.10 (0.33)	− 0.14 (0.29)	− 0.04 (0.36)	0.32^†^	0.057^†^
Change in HbA1c (mmol/mol)	Mean (SD)	− 0.40 (1.42)	− 0.25 (1.11)	− 0.57 (1.72)	0.46^†^	0.056^†^
Change in fasting insulin (mIU/L)	Mean (SD)	− 0.45 (3.82)	− 0.21 (4.04)	− 0.45 (3.41)	0.15^†^	0.43^†^
Change in ALT (µkat/L)	Mean (SD)	0.07 (0.22)	0.06 (0.28)	0.08 (0.14)	0.82^†^	0.038^†^
Change in hsCRP (mg/L)	Median (IQR)	0.00 (0.75)	0.00 (0.50)	0.00 (2.75)	0.26^‡^	0.30^‡^
Change in vitamin A (µmol/L)	Mean (SD)	0.01 (0.46)	− 0.13 (0.52)	0.17 (0.31)	0.023^†^	0.87^†^
Change in vitamin E (µmol/L)	Mean (SD)	0.51 (6.30)	− 0.04 (6.41)	1.14 (6.26)	0.54^†^	0.59^†^
Change in vitamin D (nmol/L)	Mean (SD)	1.38 (19.65)	6.42 (19.84)	− 4.38 (18.21)	0.07^†^	0.64^†^

^†^Parametric test: T-test.

^‡^Non-parametric test: Wilcoxon.

### Secondary aims

Changes in secondary aim variables and differences between groups after 3 and 6 months are provided in Tables 3 and 4 respectively.

#### BMI

After 3 months were there no differences in BMI changes between the investigated groups (p = 0.89), nor within each group (all p > 0.05). After 6 months there was a decrease in BMI from baseline to 6-month follow-up of 0.48 kg/m^2^, p = 0.027. The change in BMI did not differ statistically significant between the groups, p = 0.38. After stratification for initial probiotic dose, the group that started with double dose followed by triple dose (n = 21) decreased their BMI of 0.67 kg/m^2^, p = 0.027, whereas the group that started with single dose followed by triple dose (n = 24) decreased their BMI of 0.30 kg/m^2^, p = 0.33.

#### Waist circumference

There were no differences in change in waist circumference after 3 months between the investigated groups (p = 0.41). For those who continued with triple dose probiotics (n = 45), the waist circumference decreased from baseline to 6-month follow-up by 2.8 cm, p = 0.002.

#### Blood pressure

All groups decreased their blood pressure from baseline to 3-month follow-up, all p < 0.05, but the decrease was not different between the groups, both p > 0.75. Fort those who continues with triple dose probiotics (n = 45), the systolic and diastolic blood pressure decreased from baseline to 6-month follow-up by 6.9 mmHg and 5.8 mmHg respectively, both p < 0.0001.

#### Blood lipids

There were no changes in the levels of total cholesterol, LDL after 3 and 6 months, and no differences between the groups, all p > 0.05.

Both the placebo and the double dose probiotic group decreased the level of HDL from baseline to the 3-month follow-up with a mean (SD) of 0.10 (0.14) mmol/L, p < 0.001 and 0.11 (0.23) mmol/L, p = 0.023, respectively. However, there was no change in the level of HDL cholesterol from baseline to 6-month follow-up (− 0.03 mmol/L, p = 0.46). No differences between the groups were observed, all p > 0.05.

No group changed on average the level of triglycerides from baseline to the 3-month follow-up (all p > 0.05), and no changes between the groups were observed (p = 0.29). For those who continued with triple dose probiotics, the triglycerides decreased from baseline to 6-month follow-up by 0.22 mmol/L, p = 0.036.

#### Glucose metabolism

During the first 3 months, no group changed on average the level of fasting glucose, HbA1c or insulin from baseline, and no changes between the groups were observed (all p > 0.05). For those who continued with triple dose probiotics, there was a borderline statistically significant decrease from baseline in fasting glucose (− 0.10 mmol/L, p = 0.057) and HbA1c (− 0.40 mmol/mol, p = 0.056), whereas there were no changes in the level of fasting insulin from baseline to 6-month follow-up (− 0.45 mIU/L, p = 0.43.

#### Liver enzymes

No group changed on average the level of ALT from baseline to the 3-month follow-up (all p > 0.05), and no changes between the groups were observed (p = 0.79). At the 6-month follow-up, a minor increase in ALT levels from baseline was observed, + 0.07 µkat/L (p = 0.038). There were no differences between the groups, p = 0.82.

#### Inflammatory marker

There were no changes in hsCRP after 3 or 6 months, and no differences between the groups, all p > 0.05.

#### Vitamins

There were no changes in fat-soluble vitamins at 3-month not 6-month follow-up.

#### Blood cell composition

No changes in blood cell composition, neither between nor within groups, were observed after 3 months. After 6 months there was no change in the levels of leukocytes, mean corpuscular volume, mean corpuscular hemoglobin, or thrombocytes. However, there was a marginal decrease in the levels of erythrocytes, hemoglobin, and hematocrit. When the analyses were stratified for sex, the decrease was only observed among women (data now shown).

#### Bowel habits

Individuals who reported changes in bowel habits any time during the study were prevalent and similar between the groups; Placebo group 70.4%, single dose probiotic group 66.6%, double dose probiotic group 66.6%, triple dose probiotic group 68.9% (p = 0.99). The most common reported changes in bowel habits were changes in stool consistency and frequency of defecation. Nor were there any statistically significant differences in reported gastrointestinal problems between the groups. Frequencies of individuals that at some time reported gastrointestinal problems were 33.3% in the placebo group, 55.6% in the single dose probiotic group, 59.3% in the double dose probiotic group, and 55.6% in the triple dose probiotic group, p = 0.20. The most common reported gastro-intestinal problems included flatulence, upset stomach, and feeling of nausea.

Bowel habits were retrieved from weekly electronic questionnaires. During months four through six, all individuals responded to at least 11 out of 12–14 weekly questionnaires (depending upon follow-up date). No statistically significant changes in bowel habits or gastro-intestinal problems were observed between consumed dosage, neither for number of individuals that reported them, or number of weeks reported. Number of individuals and frequencies of reported answers are presented in Tables 5 and 6. The most common reported changes in bowel habits were changes in stool consistency and frequency of defecation. The most common reported gastro-intestinal problems included flatulence, upset stomach, and feeling of nausea.

**Table 5:** Frequency of positive answers to “Have your bowel habits changed during the last week?”.

	No. (%) of individuals*	No. (%) of responses**
Month 1–3		
Placebo group	19/27 (70.4%)	53/345 (15.4%)
Single dose probiotic group	18/27 (66.6%)	49/345 (14.2%)
Double dose probiotic group	18/27 (66.6%)	69/343 (20.1%)
Month 4–6		
All (triple dose probiotic)	31/45 (68.9%)	100/563 (17.8%)

*No statistically significant differences between the groups were detected, p = 0.99.

**No statistically significant differences between the groups were detected, p = 0.16.

**Table 6:** Frequency of positive answers to “Have you had any gastro-intestinal problems during the last week?”.

	No. (%) of individuals*	No. (%) of responses**
Month 1–3		
Placebo group	9/27 (33.3%)	30/345 (8.7%)
Single dose probiotic group	15/27 (55.6%)	33/345 (9.6%)
Double dose probiotic group	16/27 (59.3%)	42/343 (12.2%)
Month 4–6		
All (triple dose probiotic)	25/45 (55.6%)	74/563 (13.1%)

*No statistically significant differences between the groups were detected, p = 0.20.

**No statistically significant differences between the groups were detected, p = 0.65.

#### Excluded participant

One individual, a male, who received double dose AB001 during phase one, was excluded due to adverse events. He reported on day 159 that he had severe stomachache, obstipation, and concurrent vomiting. There was no gastroenteritis observed among friends and family. The study team instructed him to stop taking probiotics and thereby discontinue the study participation. He did not require any medical assistance. Four to five days after stop taking probiotics, he reported that he had a normal gastrointestinal function again. As a safety precaution he was taken back for a check-up. Approximately 1 month after not taking the probiotic compound he had no gastrointestinal symptoms. From baseline, the individual had lost 2.3 kg in weight, corresponding to a decrease in BMI of 0.69 kg/m^2^. Overall, the biochemical profile remained unchanged. At follow-up, CRP remained low (1 mg/L). Total cholesterol and LDL were borderline high (6.1mmol/L and 4.4 mmol/L respectively). HDL, triglycerides, glucose, insulin, HbA1c, and ALT remained normal.

## Discussion

In this study, we assess the impact of the probiotic compound AB001 on weight loss among individuals with overweight. During the initial 3-month double-blinded placebo-controlled phase, no difference in weight was observed between the groups using placebo, single dose, or double dose of the compound. During the second phase, individuals who were administrated a, high dose probiotic, i.e. first double and then triple dose, experienced a statistically significant weight reduction from their baseline of approximately 2 kg.

Some probiotic compounds have demonstrated an advantage over placebos in facilitating weight loss among both adult and pediatric populations with excessive weight, suggesting a potential beneficial role of probiotics in weight management strategies^7,8,16,17^. It should be noted, though, that this outcome is not a consensus, as some studies have reported contrasting findings^18^. In the present study, a modest reduction in weight was observed from baseline to the 6-month follow-up among participants who completed the full study. Even though it is possible that the provided pamphlet of dietary habits may have contributed to this effect, there is a possibility that an association exists between AB001 dosage and time, indicative of a potential dose–response relationship. This is supported by the fact that the impact on weight (and BMI) was more pronounced in cases where individuals consumed a double dose, followed by a triple dose, over an additional 3-month period. The observed weight reduction, though statistically significant, is below the clinically relevant threshold of an annual decrease of 5%, known to enhance cardiometabolic health in individuals with obesity^19^. Notably, our study focused on otherwise healthy overweight participants over 6 months, potentially impacting the magnitude of observed effects.

Probiotics, whether residing in the gut or passing through, have diverse effects on the host. They modulate gut functions, bolster immune responses, influence metabolism, and regulate food intake signals^20^. Interestingly, probiotics' interactions with host epithelial cells can lead to beneficial changes in their behavior, enhancing their symbiotic relationship with the host^20^. *Bacillus coagulans*, which is one of the major strains in AB001, has been shown to improve the utilization of consumed foods, mainly due to its capacity to produce a range of enzyme, such as lipases and galactosidases^21^. Individuals with a normal weight harbor a distinct gut bacterial composition compared to those with overweight or obesity, which may be of importance for the understanding of the variable effects observed in different studies^22,23^. The modulation of the bacterial strains in the digestive tract may help to reshape the metabolic profile in humans with obesity as suggested by several data from animal and human studies^20,23^. Moreover, the effectiveness of probiotic supplements in facilitating weight loss has been linked with specific dosages and types of probiotics, highlighting that the beneficial impacts are not universally observed and may depend on several factors including the choice of probiotic strains^24–26^. Thus, further investigations into the dose-dependency and strain-specific effects of probiotics are warranted to elucidate their potential role in managing obesity and optimizing metabolic health.

The aim of the second phase was primarily to evaluate safety and potential side effects. Potential harm of probiotics is rarely discussed, and it is therefore warranted to investigating side effects of probiotic supplementation^20,28^. We could not observe any gastro-intestinal harm of increased probiotic dose. About 2/3 reported changes in bowel habits and between 55 and 60% reported intermittent gastro-intestinal problems regardless of probiotic dose or placebo consumed. Given that the triple dose was preceded with either single or double dose of AB001, we cannot exclude that there was an adaptation to the higher consumed dose of probiotics. One individual reported severe stomach pains after 5 months, but whether it was caused by the tested compound is unclear as no similar signs were observed among the other participants.

The compliance and adherence to the study protocol was very good. The study coordinator had regular contact with the participants via the digital support system and as the weekly weight measurements were automatically transferred to the database. Via the study app reminders could be sent out if data was missing. Together this probably contributed to the low dropout rate.

The effect of probiotics on biomarkers have been investigated with mixed findings^16,21^. We found some beneficial changes of clinical investigations and biomarkers. Both systolic and diastolic blood pressure decreased over the six investigated months. Further, in the total sample a minor, yet statistically significant decrease in triglycerides was observed. Both fasting glucose and HbA1c decreased with borderline statistical significance in the full sample. A slight increase in ALT was observed over the studied period. If the observed changes are due to the probiotic supplement or secondary to lifestyle changes remains to be evaluated in a long-term high dose placebo controlled clinical trial.

The minor changes in erythrocytes, hemoglobin and hematocrit were only observed among women. Since other biomarkers of diet and inflammation (e.g. lipid profile, vitamins, and CRP) remained normal, it is likely that the observed minor change was due to physiological alterations caused by menstruation.

Even though the study was well conducted, there are some limitations that should be acknowledged. We did not collect any objective measures of mediators for weight gain or weight loss, e.g. amount of physical activity or dietary changes. However, we did collect objectively measured weight throughout the study. Further, despite the relatively large sample size, the heterogeneity prevented us from making post-hoc analyses to investigate potential effects in subgroups, e.g. having heredity for overweight or obesity. Moreover, due to the study design allowing participant continuation of probiotics from the initial phase, randomization unblinding took place before data analysis. The robust nature of the exclusion criteria, such as the deliberate exclusion of weight-related comorbidities, might potentially limit the extent to which these findings can be extrapolated to a broader population.

## Conclusion

In this trial testing probiotics for weight loss, no effect was observed after the double-blinded 3-month phase. For those who received double dose for 3 months who continued with triple dose for another 3 months in the open study phase, a modest but significant weight loss of approximately 2 kg or 0.7 BMI units was observed. A reduction in blood pressure was also noted. The current study provides evidence that a double and triple dose of the probiotic compound AB001 is well tolerated and can be consumed safely by adults. Further studies are required to certify to which extent this compound can facilitate weight loss and metabolic benefits.

### Supplementary Information

Supplementary Information 1.

Supplementary Information 2.

## Data Availability

The datasets generated and analyzed during the current study are not publicly available due to lack of informed consent, but de-identified datasets are available from the corresponding author on reasonable request.
