# Supplementary material for: Evaluating probiotic efficacy on weight loss in adults with overweight through a double-blind, placebo-controlled randomized trial
Source: Sci Rep. 2023 Oct 24;13:18200. doi: 10.1038/s41598-023-45395-7 (PMC10597999; doi:10.1038/s41598-023-45395-7)
Supplement: Supplementary file 1 — Supplementary Information 1. [file 41598_2023_45395_MOESM1_ESM.pdf]

Background, physical activity, and dietary habits

1. **Do you consider yourself healthy?** ◻ **Yes** ◻ **No**

   If no, please descibe...

   ....

2. **Have you had Covid-19 or are you vaccinated?** ◻ **Yes** ◻ **No**

3. **Have you experienced any prior illnesses that necessitated medical treatment or hospital care?**

   ....

   ....

4. **Do you have any allergies? If so, what are they in reaction to?**..........

   ....

5. **Are you currently using any medications, dietary supplements, or vitamins?**

   If yes, what are you using, and at what dosage?.......................................

   ....

6. **In which country were you born in?**

   ....

7. **What is your occupation?** Please indicate with only one checkmark (✓).
   - ☐ I work
   - ☐ I'm a student
   - ☐ I'm between jobs
   - ☐ I'm on parental leave

8. **What is your family situation?** Feel free to use as many checkmarks (✓) as necessary to indicate your situation.
   - ☐ I live alone
   - ☐ I live with a partner
   - ☐ I live with my mother and/or father
   - ☐ Other:…………………………………………………………..

9. **Physical activity?** Please indicate with only one checkmark (✓).

□ I have a fairly low activity
□ I move, but never to the point where I'm out of breath and sweaty
□ I move to the point where I'm out of breath and sweaty sometimes
□ I move to the point where I'm out of breath and sweaty several times weekly
□ I move to the point where I'm out of breath and sweaty every day or almost every day

10. **Nicotine and tobacco habits.** Please indicate with only one checkmark (✓).

☐ I'm a daily user
☐ I use it sometimes
☐ I never use nicotine or tobacco

11. **Bowel habits.** Please indicate with only one checkmark (✓).

Do you have regular visits to the restroom? □ **Yes** □ **No**

How often?

☐ Several times daily
☐ Once daily
☐ A few times weekly
☐ Less than once weekly

Appearance?

☐ Without remarks or any problems
☐ Soft
☐ Hard
☐ Painfully hard

Thank you for taking the time to answer the questions!
