# Supplementary material for: Evaluating probiotic efficacy on weight loss in adults with overweight through a double-blind, placebo-controlled randomized trial
Source: Sci Rep. 2023 Oct 24;13:18200. doi: 10.1038/s41598-023-45395-7 (PMC10597999; doi:10.1038/s41598-023-45395-7)
Supplement: Supplementary file 2 — Supplementary Information 2. [file 41598_2023_45395_MOESM2_ESM.pdf]

Translated. [Translator’s note]

WEEKLY QUESTIONNAIRE IN THE MOBILE APPLICATION

General condition

Have you during the last week had to stay home from work or school due to feeling unwell?

- No
- Yes

Have you taken any medicines/dietary supplements in addition to those reported at the start?

- No
- Yes [if ticked, the following questions arose]
  - Headache Relief Tablets (e.g. Alvedon [paracetamol], Ipren [ibuprofen], TREO [acetylsalicylic acid]).
    - [If ticked] How many?
  - Antibiotics.
    - [If ticked] How many days?
  - Vitamines
    - [If ticked] Type?
    - [If ticked] How many?
  - Dietary supplement
    - [If ticked] Type?
    - [If ticked] How many?
  - Other
    - [If ticked] Please descibe [free text]

Diet

Have you changed your dietary habits during the last week?

- No
- Yes [if ticked, the following questions arose]
  - I eat less
  - I eat more
  - I eat healthier than usual
  - I eat less healthier than usual

Physical activity

Have your physical activity habits changed during the last week?

- No
- Yes [if ticked, the following questions arose]
  - More physically active
  - Less physically active

Study adherance

Have you taken all your probiotic capsules during last week?

- Yes
- No [if ticked, the following questions arose]

    - How many have you missed?
        - Less than 5 capsules
        - 5 to 10 capsules
        - More than 10 capsules

Bowel habits and stomach

Have your bowel habits changed during the last week?

- No
- Yes [if ticked, the following questions arose]
    - More frequent defecation
    - Less frequent defecation
    - More irregular defecation
    - Harder stool
    - Looser stool
    - Changed color

Have your stomach felt differently during the last week?

- No
- Yes [if ticked, the following questions arose]
    - Increased gas production (more than usual)
    - I'm experiencing a form of bloating that feels unfamiliar compared to before.
    - My stomach is more upset than usual
    - I have been experiencing nausea.
    - I have experienced unexplained episodes of vomiting.

The weekly questionnaire is now complete. Thank you for your participation.
